# Kinetic and High-Pressure Mechanistic Investigation of the Aqua Substitution in the *Trans-*Aquaoxotetracyano Complexes of Re(V) and Tc(V): Some Implications for Nuclear Medicine

**DOI:** 10.1155/2008/745989

**Published:** 2008-04-17

**Authors:** J. Mattheus Botha, Andreas Roodt

**Affiliations:** ^1^Department of Chemistry, University of the Orange Free State, P.O. Box 339, Bloemfontein 9300, South Africa; ^2^Sasol Technology R & D, P.O. Box 1, Sasolburg 1947, South Africa

## Abstract

A kinetic study of the aqua substitution in the [TcO(OH_2_)(CN)_4_]^−^ complex by different thiourea ligands (TU = thiourea, NMTU = *N*-methyl thiourea, NNDMTU = *N*, *N*′-dimethylthiourea) yielded second-order formation rate constants (25°C) as follows [NNDMTU, NMTU, TU, respectively]: *k_f_* = 11.5 ± 0.1, 11.38 ± 0.04, and 7.4 ± 0.1 M^−1^s^−1^, with activation parameters: Δ *H^#^_k_f__* : 55 ± 2, 42 ± 3, 35 ± 5 kJ mol^−1^; Δ*S^#^_k_f__* : − 40 ± 8, − 84 ± 11, − 110 ± 17 J K^−1^mol^−1^. A subsequent high-pressure investigation of the aqua substitution in the [ReO(OH_2_)(CN)_4_]^−^ and [TcO(OH_2_)(CN)_4_]^−^ complexes by selected entering ligands yielded Δ*V^#^_k_f__* values as follows: Re(V): −1.7 ± 0.3(NCS^−^), −22.1 ± 0.9 (TU) and for Tc(V): −3.5 ± 0.3(NCS^−^), −14 ± 1 (NNDMTU), and −6.0 ± 0.5 (TU) cm^3^mol ^−1^, respectively. These results point to an interchange associative mechanism for the negative NCS^−^ as entering group but even a pure associative mechanism for the neutral thiourea ligands.

## 1. INTRODUCTION

Technetium-99m is widely used in over 90% of all current diagnostic nuclear medicinal applications [1–5] due to its favourable nuclear properties (*t*_1/2_ = 6.02 hours, *y* = 140 keV 100%) and availability from a generator. It is routinely used for brain, heart, liver, kidney, and bone imaging. Technetium’s third-row congener and 5d analog, rhenium, also has applications in nuclear medicine since its two radioactive isotopes, Re-186 and Re-188, have nuclear properties suitable for radiotherapeutic applications [6–8].

The development of technetium myocardial imaging agents commenced with work by Deutsch [9–12], who investigated the [^99m^TcCl_2_(dmpe)_2_]^+^ [dmpe = bis(1,2-dim ethylphosphino)ethane] and [^99m^TcX_2_(diars)_2_]^+^ complexes (diars = *o*-phenylenebis(dimethylarsine), *X* = Cl, Br) [12]. Studies on animal models indicated that the reduction of Tc^III^ is biologically accessible for the cationic [^99m^Tc^III^–Cl_2_(dmpe)_2_]^+^ complex, yielding neutral [^99m^Tc^II^Cl_2_(dm- pe)_2_]. The latter then washes from the heart and becomes trapped in the liver. The reduction of Re^III^ to Re^II^ [^186^Re^III^ – Cl_2_(dmpe)_2_]^+^ is 0.2 V more negative compared to the anal- ogous Tc complex and is thus retained in the heart [10]. Kinetic, electrochemical, and structural work on the [Re/ Tc^II^Cl_2_(dmpe)_2_] complexes has been published and illustrates its importance as initial models [13–18].

Currently, however, other monocationic complexes of technetium-99m are of significant interest because of their extensive use as ^99m^Tc myocardial imaging agents [19, 20] with examples including *Cardiolite* or [^99m^Tc(MIBI)_6_]^+^ (MIBI = 2-methoxy-2-methylpropylisocyanide) [21, 22] and *Myoview* [^99m^TcO_2_(Tetrofosmin)_2_]^+^. While the monoca tionic complexes are traditionally based on the [O=Tc^V^ =O]^+^, [Cl–Tc^III^–Cl]^+^, and Tc^I^ cores, a new class of myocardial im- aging agents feature the [^99m^Tc^V^≡N]^2+^ core, an exam- ple being the Tc–N–NOET complex (*bis*(*N*-methoxy-*N*- methyldithiocarbamato)nitridotechnetium(V)).

Two technetium complexes, containing specifically *phosphine* ligands, currently used for myocardial imaging are the above-mentioned *Myoview* and *TechneScan* *Q12/TechneCard* ([^99m^Tc(PR_3_)_2_(L′)]^+^) [23]. In [^99m^TcO_2_(Tetrofosmin)_2_]^+^ the technetium is in a high-oxidation state {Tc(V)} and shows substantial myocardial uptake [24]. It is interesting to note that [^99m^TcO_2_(dmpe)_2_]^+^, which has methyl groups on the phosphine rather than the ether groups as in the tetrofosmin ligand, is not retained in the myocardium [25].

With the ongoing development of new Tc and Re agents, it is essential that their basic coordination chemistry is understood. Nonradioactive rhenium is widely utilized to imitate technetium chemistry on a macroscale and has been extensively pursued for the past two decades or more, describing changes in coordination modes. We reported structural effects induced by different Re-cores while maintaining an equatorial ligand set, utilizing two dmpe ligands, while varying the axial core, to investigate the impact this change induces on the solid-state structure of the coordinated polyhedron and on the bidentate tertiary phosphine ligand, dmpe [26]. Similarly, the effect of different conformers/isomers and energies associated therewith has been described [27]. More recent extensive work by Alberto showed that the *fac*-[(CO)_3_M(H_2_O)_3_]^+^ {M = Re(I), Tc(I)} core provides excellent access to numbers of model radiopharmaceuticals [28–35].

The “lanthanide contraction” results in similar physical characteristics for analogous Re and Tc complexes (i.e.*,* size or lipophilicity) [1, 6]. Thus, when rhenium is used as the nonradioactive surrogate for the development of Tc chemistry because it is nonradioactive, their similar physical characteristics make it very difficult for biological systems to distinguish between analogous Tc and Re [6] complexes based on properties such as size, shape, and charge. However, firstly, they differ significantly in their redox properties, which can result in different in vivo handling of analogous complexes. Rhenium complexes are more stable in higher-oxidation states and thus are more difficult to reduce (by ca. 200 mV) than their Tc analogs [36]. Thus, Re is more readily reoxidized to perrhenate (ReO^−^_4_) than Tc is to pertechnetate (TcO_4_^−^) in vivo*,* and perrhenate requires the use of stronger reducing agents for the synthesis of Re radiopharmaceuticals.

A second difference is the larger ligand field splitting for Re complexes, which results in slower-ligand substitution onto Re than Tc. We have previously investigated different aspects of the *trans-*dioxo complexes, with the general structure of *trans*-[MO_2_(L_4_)]^*n*−^, M = Mo(IV), W(IV), Tc(V), Re(V), Os(VI), and related systems, evaluating structural and reactivity correlations for a range of ligands L. It was shown that a twelve-order of magnitude in reactivity in these systems exists, ranging from the very rapid proton exchange, to the slower hydroxo and aqua substitution and the extremely slow-equatorial ligand substitution [37–41].

Subsequent kinetic studies on the [ReO(OH_2_)(TU)_4_]^3+^ complex, showed that the *trans-*substitution reactions of the aqua ligand most likely proceed via an interchange dissociative mechanism (*I_d_*) [42]. This outlined a discrepancy in the proposed mechanism for *trans-*substitution reactions on the [ReO(OH_2_)(CN)_4_)^−^ complex as concluded earlier in the literature [43, 44]. The substitution rate for the [ReO(OH_2_)(TU)_4_]^3+^ complex with NCS^−^ was in the order of ca. 450 000 times faster than for the [ReO(OH_2_)(CN)_4_]^−^ complex. Furthermore, the reactions involving [ReO(OH_2_)(TU)_4_]^3+^ and higher concentrations of the entering ligand showed typical limiting kinetics, while in the [ReO(OH_2_)(CN)_4_]^−^ complex, no tendency of limiting kinetics was observed.

We have previously correlated different in vivo reactivities with in vitro behaviour [41] and attempted to link certain sites with biodistribution and bioactivity, but was, and still is, unable to do more detailed comparisons. Thus, since detailed mechanistic studies and data on substitution processes are fairly limited, it prompted us to reinvestigate the type of mechanism obeyed for the [ReO(OH_2_)(CN)_4_]^−^ complex when reacted with different entering ligands and extending it to the Tc^V^ complex, by specifically utilizing advanced high-pressure kinetics. This high-pressure study of the [MO(OH_2_)(CN)_4_]^−^ complex (M = Re and Tc), with different entering ligands, is therefore reported here.

## 2. MATERIAL AND METHODS

All reagents and chemicals were of analytical reagent grade, and double-distilled water was used in all experiments. All pH measurements were done with an Orion 701 pH meter and a combined glass/calomel electrode using standard buffer solutions and standardized hydrochloric acid solutions for calibration. The ionic strength was constant (*μ* = 1M) in all the experiments, maintained with NaNO_3_ as noncoordinating electrolyte. In all calculations, pH = −log [H^+^]. Na_3_[ReO_2_(CN)_4_] and Na_3_[TcO_2_(CN)_4_] were prepared as previously described [37]. *Caution:* Technetium-99, although a low-energetic radio-active *β*-emitter (230 KeV, *t*_1/2_ = 2.1 × 10^5^ *y*), should always be handled with care and under approved conditions.

Kinetic measurements were done on modified Durrum-Gibson Model D110 and Applied Photophysics SX.18 MV (control experiments; coupled with a J&M Tidas-16 diode array) stopped flow spectrophotometers equipped with constant temperature syringe and cell holder systems (accurate within 0.1°C). These were coupled to a personal computer or Acorn Risc workstation capable of performing least-squares analyses on the absorption values versus time data obtained from the kinetic runs. The SCIENTIST [45] program was used to fit the data to selected functions. High-pressure studies were done on a GBC 916 spectrophotometer in a high-pressure vessel with pill box cells of path length *≈*15 mm or in a stopped flow high-pressure vessel [46]. All kinetic runs were performed under pseudo-first-order conditions with the ligand in large excess. The solid lines in the figures represent computer least-squares fits of data, while the experimentally determined values are given as points. The [MO(L)(CN)_4_]^*n+*^ complexes from the reactions between [MO(OH_2_)(CN)_4_]^−^ and different entering ligands were characterised as previously described [43, 44].

## 3. RESULTS AND DISCUSSION

It was previously shown that the complete reaction scheme governing the substitution reactions on the protonated forms of the *trans*-[MO_2_(CN)_4_]^(*n+2*)^−^^ complexes is limited to the aqua species, *trans*-[MO(OH_2_)(CN)_4_]^*n*−^ and with small con tributions, under selected conditions from *trans*-[MO(OH)- (CN)_4_]^(*n*+1)^−^^ [37]. Assumptions made and approximations have all been reported previously.

**Scheme 1:**
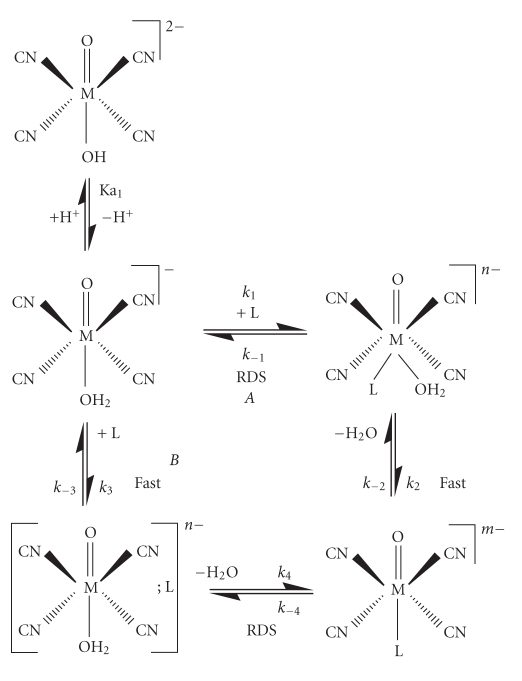
Illustration of an associative (*A*; *k*_1_/*k*_2_) or associative interchange (*B*; *k*_3_/*k*_4_). Activation for the aqua substitution in [MO(OH_2_)(CN)_4_]^−^[M = Re(V), Tc(V)]; RDS = rate determining step.

**Figure 1:**
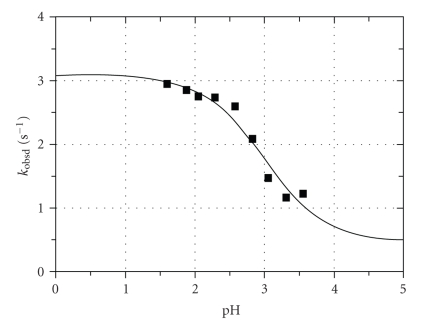
Plot of *k*_obsd_ *versus* pH for the reaction between [TcO(OH_2_)(CN)_4_]^−^ and NNDMTU at 24.8°C, *μ* = 1.0 M(NaNO_3_), [NNDMTU] = 0.3 M, and [M] = 5 × 10^−5^ M, *λ* = 420 nm.

The *pK*_*a*1_ value (of the [TcO(OH_2_)(CN)_4_]^−^ complex, Scheme 1) was previously determined from the reaction between [TcO(OH_2_)(CN)_4_]^−^ and NCS^−^ ions as 2.90(5) by Roodt et al. [44]. To further verify this by another ligand system, an independent kinetic *pK*_*a*_ determination was carried out for the reaction between [TcO(OH_2_)(CN)_4_]^−^ and NNDMTU and is illustrated in Figure 1. NNDMTU was selected since these reactions showed the largest absorbance changes.

The general expression for the observed pseudo-first-order rate constant {[L] ≫ [M]} shown in (1), as obtained previously, describes the acid-base behaviour of the *trans*-[MO(OH_2_)(CN)_4_]^*n*−^ complexes, where *k_f_* and *k_r_* represent the forward and reverse rate constants, that is, the anation/ligation and acid hydrolysis, respectively.

(1) $$k_{\text{obsd}}=\frac{k_{f}\left[\text{L}\right]}{1+K_{a_{1}}/\left[{\text{H}}^{+}\right]}=k_{r}.$$

The data in Figure 1 was fitted to (1), and a *pK*_*a*1_ value as reported in Table 1 was obtained. The acid dissociation constant thus determined from the reaction between [TcO(OH_2_)(CN)_4_]^−^ and NNDMTU {2.99 ± 0.19} is in good agreement with the *pK*_*a*_ value reported for the reaction between the metal complex and NCS^−^ ions (2.90 ± 0.05) [44].

It is therefore evident that if the reaction between [TcO(OH_2_)(CN)_4_]^−^ and the different entering ligands (TU, NMTU, NNDMTU) is investigated at a pH value of 0.6 [*pK*_*a*1_ > 2.9, see Table 1], the *trans*-oxo aqua species of the metal complex is more than 99% present in solution. At these high-acidic conditions where *K*_*a*1_ ≪ [H^+^], (1) simplifies to the well-known simple expression in (2), assuming negligible reverse or concurrent reactions (*k_r_* ca. 0). The *k*_obsd_ versus [L] data obtained from these runs was fitted to (2), and values for *k_f_* and *k_r_* were consequently obtained (Table 1):

(2) $$k_{\text{obsd}}=k_{f}\left[\text{L}\right]+k_{r}\approx k_{f}\left[\text{L}\right].$$

**Figure 2:**
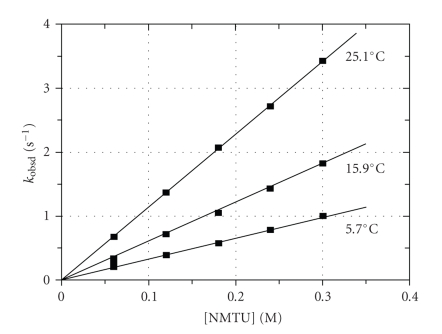
Effect of *k*_obssd_ versus [NMTU] for the reaction between [TcO(OH_2_)(CN)_4_]^−^ and NMTU at different temperatures, *μ* = 1.0 M (NaNO_3_), *λ* = 420 nm, pH = 0.6, and [M] = 5 × 10^−5^ M.

The ligand concentration and temperature dependence study for each of the different thiourea entering ligands (TU, NMTU, and NNDMTU) were therefore completed for the [TcO(OH_2_)(CN)_4_]^−^, with the data for NMTU as entering ligand shown in Figure 2.

These *k_f_* versus temperature data sets were used in the Eyring equation [43] to calculate the activation parameters governing the *k_f_* step (Table 1). The intercepts (*k_r_*) in all these runs were zero within standard deviations, thus confirming a large *k_f_* value (*K_f_* = (*k_f_*/(*k_r_*) for each of the different nucleophiles.

The activation entropies (Table 1) for all the reactions studied suggest increased order in the transition state, indicative of association being important.

Following similar arguments used by Grundler et al. [35], and by our group [47], different pathways for the substitution process were therefore considered.

Firstly, for an *associative* mechanism (Scheme 1, *A*, *k*_1_ and *k*_2_ pathway), the rate of the reaction is given by

(3) $$\text{Rate}=k_{2}[\text{MO}({\text{OH}}_{2})(\text{L}){{\left(\text{CN}\right)}_{4}}^{n-}]-k_{-2}[\text{MO}(\text{L}){{\left(\text{CN}\right)}_{4}}^{m-}].$$

If it is assumed that the [MO(OH_2_)(L)(CN)_4_]^*n*^ complex is formed under steady-state conditions, its formation and decomposition would be equal yielding

(4) $$[\text{MO}({\text{OH}}_{2})(\text{L}){{\left(\text{CN}\right)}_{4}}^{n-}]=\frac{k_{1}\left[\text{MO}({\text{OH}}_{2}){{\left(\text{CN}\right)}_{4}}^{-}\right]\left[\text{L}\right]+k_{-2}\left[\text{MO}(\text{L}){{\left(\text{CN}\right)}_{4}}^{m-}\right]}{k_{-1}+k_{2}}.$$

Upon incorporation of the definition of *K*_*a*1_ (= [MO(OH) (CN)_4_^2−^][H^+^]/[MO(OH_2_)(CN_4_^−^)]), [M]_tot_ (= [MO(OH_2_) (CN)_4_^2−^] + [MO(OH)(CN)_4_^2−^]) and substituting (4) into (3), integration of the rate law {[L] ≫ [M]}, by assuming a fast *k*_2_ step (Scheme 1), 5 {with *k*_2_′ = *k*_1_ *k*_2_/(*k*_−1_ + *k*_2_)}, and defining the pseudo-first-order rate constant, is obtained:

(5) $$k_{\text{obsd}}=\frac{{k_{2}}^{\prime }\left[\text{L}\right]}{1+{K_{a_{1}}}_{}/\left[{\text{H}}^{+}\right]}+k_{-2}.$$

Similar arguments may be used, considering an *interchange* pathway (Scheme 1, *k*_3_ and *k*_4_), incorporating the definition of *K*_*a*1_ (= [MO(OH)(CN)_4_^2−^][H^+^]/[MO(OH_2_)(CN)_4_^−^]), [M]_tot_ (= [MO(OH_2_)(CN)_4_^−^] + [MO(OH)(CN)_4_^2−^] + [MO(OH_2_)(L)(CN)_4_^*n*−^]), *K*_3_(= [MO(OH_2_)(L)(CN)_4_^*n*−^]/[MO(OH_2_)(CN)_4_^−^][L]; ([L] ≫ [M]_tot_), yielding an expression for the pseudo-first-order rate constant as given in (6), and assuming *K*_3_[L] ≪ 1,

(6) $$k_{\text{obsd}}=\frac{k_{4}K_{3}\text{[L]}}{K_{a_{1}}/[{\text{H}}^{+}]+\text{1}}+k_{-4}.$$

It is clear that (5) and (6) are similar and both adequately describe the experimental results { *associative* mechanism (5): *k_f_* = *k*_2_′ and *k_r_* = *k*_−2_;*interchange* mechanism (6): *k_f_* = *k*_4_ *K*_3_ and *k_r_* = *k*_−4_ and *K*_3_ = *k*_3_/*k*_−3_}, and both simplify to (1) and (2), respectively.

The pressure dependence for the substitution process as studied here, at different pressures *a* and *b*, is given by [47]

(7) ln(ka/kb)=−ΔV#expt.(Pa−Pb)/RT.

**Figure 3:**
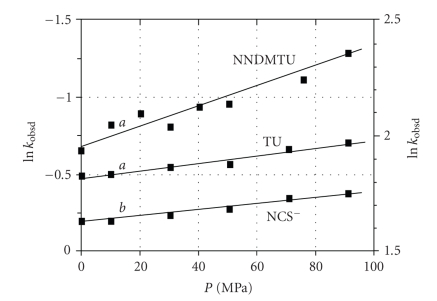
The effect of pressure on the second-order formation rate constant for the reaction between [TcO(OH_2_)(CN)_4_]^−^ and NCS^−^, NNDMTU, and TU at 25°C, pH = 0.6, [M] = 5 × 10^−5^ M, *λ* = 420 nm, *a* refers to left axis, *b* refers to the right axis. [NCS^−^] = 0.2 M, [NNDMTU] = [TU] = 0.05 M, *μ* = 1.0 M (NaNO_3_).

Since the contribution by the reverse step is negligible in all cases in this study as concluded above, this implies that Δ*V^#^*_expt_ ≈ Δ*V^#^*_*k_f_*_. The data obtained for the *trans*-[TcO (OH_2_)(CN)_4_]^−^ are shown in Figure 3, where (7) was utilized to obtain Δ*V^#^*_expt_, and the results are reported in Table 1.

In order to compare the type of mechanism obeyed for *trans-*aqua substitution in the [ReO(OH_2_)(CN)_4_]^−^ complex, a high pressure study with NCS^−^ ions and TU was also performed. Since the reaction between [ReO(OH_2_)(CN)_4_]^−^ and L (L = NCS^−^ and TU) shows equilibrium constants of 87 ± 7 and 7.0 ± 0.4 M^−1^, respectively [48, 49], similar arguments to the Tc(V) as mentioned above could be used to determine the activation volume, Δ*V^#^*_expt_, for which the values are reported in Table 2.

**Figure 4:**
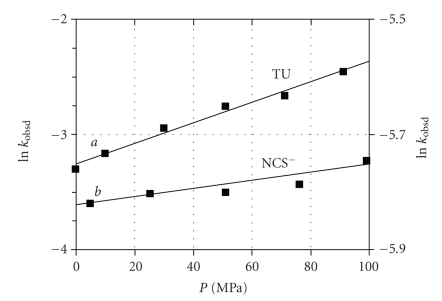
The effect of pressure on the second-order formation rate for the reaction between [ReO(OH_2_)(CN)_4_]^−^ and NCS^−^ ions and TU at 25°C, pH = 0.3, [M] = 1.5 × 10^−3^ M, *a* refers to left axis, *b* refers to right axis. [NCS^−^] =0.6 M, *λ*_NCS_^−^ = 420 nm; [TU] = 1.0 M, *λ*_TU_ = 350 nm, *μ* = 1.0 M (NaNO_3_).

This high pressure kinetic study on the reaction between [TcO(OH_2_)(CN)_4_]^−^ and NCS^−^ ions, NNDMTU and TU [Figure 3], yields Δ*V^#^*_expt_ values of −3.5 ± 0.3, −14 ± 1 and −6.0 ± 0.5 cm^3^/mol, respectively. Similarly, the data for the Re(V), as represented in Figure 4 gave Δ*V^#^*_expt_ values of −1.7 ± 0.3 and − 22.1 ± 0.9 cm^3^/mol for NCS^−^ ions and TU respectively. It is clear that all these indicate small to large negative values, contrary to the experiments on Alberto's Re^I^ and Tc^I^ complexes [35].

We previously concluded that with regard to the mechanism, due to the large distortion (metal displaced out of the plane formed by the four *cis* ligands bonded to the metal, away from the *trans*-oxo) observed for the [MO(L)(CN)_4_]^*n*−^ complexes of Mo^IV^, W^IV^, Re^V^, and Tc^V^, a dissociative acti- vation would be favoured during *trans-*aqua substitution reactions [44, 47]. A positive volume of activation (+10.6 ± 0.5 cm^3^/mol) was observed for the reaction between the corresponding isostructural [WO(OH_2_)(CN)_4_]^2−^ complex and N^−^_3_, forming an important basis of the mechanistic assignment.

However, the current high-pressure study on the [ReO(OH_2_)(CN)_4_]^−^ and [TcO(OH_2_)(CN)_4_]^−^ complexes clearly indicates a negative volume of activation for all these reactions (Table 2), ranging from slightly negative for the anionic NCS^−^ as entering ligand, to substantially negative for the neutral thiourea ligands. This yields important evidence, along with the large negative Δ*S^#^* values, that an associative (*A*) mechanism or an associative interchange (*I_a_*) mechanism is operative for the activation step during these *trans-*aqua substitution reactions on the M(V) metals. In principle, this is actually quite acceptable, since the [MO(OH_2_)(CN)_4_]^*n*−^ complexes of Mo^IV^, W^IV^, Re^V^ and Tc^V^ are all classic 16 electron species. Clearly, the M(IV) metal centres are softer than the corresponding Re^V^ and Tc^V^, allowing easier dissociation of the aqua ligand in the rate determining step. This is confirmed by the solid state structures of the [MO(NCS)(CN)^4^]^2−^ complexes, wherein both of the NCS^−^ ligands where nitrogen bound. [43, 44].

The formation of the [MO(L)(CN)_4_]^*m*−^ complex in Scheme 1 in an *A* mechanism yields an activation volume Δ*V^#^*_expt._ = Δ*V^#^_k_f__* = Δ*V^#^*_*k*__2_, which is expected to be large negative, and holds true for the reactions between both [ReO(OH_2_)(CN)_4_]^−^ and TU (Δ*V^#^*_*k*__2_′ = −22.1 (9)  cm^3^ mol^−1^) and [TcO(OH_2_)(CN)_4_]^−^ and NNDMTU (Δ*V^#^_k__f_* = −14(1) cm^3^ mol^−1^) and to a lesser extend for [TcO (OH_2_)(CN)_4_]^−^ and TU (Δ*V^#^_k__f_* = −6.0 ± 0.5 cm^3^ mol^−1^). It is therefore realistic that the neutral ligands will associate more easily with the [MO(OH_2_)(CN)_4_]^−^ species, compared to association between two negative species {[MO(OH_2_)(CN)_4_]^−^ and the NCS^−^ ligand}, supporting the assumption of an associative process. Furthermore, the reaction between [MO(OH_2_)(CN)_4_]^−^ and NCS^−^ yielded activation values of −1.7 ± 0.3 and −3.5 ± 0.3 cm^3^ mol^−1^ for Re^V^ and Tc^V^, respectively. These are considered too small negative values to support a pure associative activation, although electrostriction between the negatively charged complex and entering NCS^−^ ligand might affect the total value of Δ*V* ^#^_expt._.

For an *I_a_* mechanism, the volume of activation can be expressed as the sum of the individual contributions for each step in Scheme 1 (8), where *k_f_* = *k*_4_ *K*_3_ and Δ*V*_*K_3_*_ = reaction volume for the equilibrium reaction defined by *K*_3_:

(8) $${{\Delta V}^{\#}}_{\text{expt.}}={\Delta V}_{K_{3}}+{{\Delta V}^{\#}}_{k_{4}}.$$

The *k*_4_ step is associated with a simultaneous bond breaking/formation process, and therefore Δ*V* ^#^_*k*_4__ is expected to be slightly negative in an interchange associative process. Furthermore, Δ*V*_*K*_3__ can be expressed in terms of its individual components (9):

(9) $${\Delta V}_{K_{3}}={{\Delta V}^{\#}}_{k_{3}}-{{\Delta V}^{\#}}_{k_{-3}}.$$

Since Δ*V* ^#^_*k*_−3__ is expected to be positive (associated with bond breaking), and Δ*V* ^#^_*k*_3__ in turn is slightly negative, Δ*V*_*K*_3__ is expected to be either small positive or slightly negative. It is thus clear from (9), that depending on the relative magnitude of the volume change associated with *K*_3_ and *k*_4_, that either an *I_d_* or *I_a_* mechanism is possible. However, since an overall negative tendency for Δ*V* ^#^_expt._ was obtained, an associative interchange mechanism is considered more likely for the NCS^−^ reaction, since there should be significant electrostriction between the NCS^−^ and [M]^−^ species. For the neutral thiourea ligands, an even larger-negative activation volume is observed, and a pure associative mode of activation could well be operative.

Upon comparison of the *trans-*substitution reactions on the [ReO(OH_2_)(CN)_4_]^−^ and [TcO(OH_2_)(CN)_4_]^−^ complexes (Table 2), a few other interesting observations are also made.

Firstly, for the [ReO(OH_2_)(CN)_4_]^−^ complex, the order of reactivity for the ligands are: NMTU > NNDMTU > TU > NCS^−^> HN_3_ and for the [TcO(OH_2_)(CN)_4_]^−^ complex: NCS^−^> NNDMTU > NMTU > TU. It is clear that the NCS^−^ ligand shows the fastest reaction with the Tc centre (ca. 6300 times faster), but the slowest reaction with the Re centre [see relative ratio’s of *k_f_* Tc/*k_f_* Re in Table 2]. A comprehensive explanation of this rate difference is not yet possible. It is, however, known that the Tc^V^ centre can more readily accept electron density than does the Re^V^ [50, 51] and to some extend favour, in spite of the negative charge on the NCS^−^ as entering ligand, association with the Tc^V^ centre. This is, however, not manifested in the activation volumes. The rate constants of the thiourea ligands are more comparable, showing a great deal of consistency for both metal centres, although a slight dependence on steric bulk/electron density of the TU ligands is apparent, but cannot currently be convincingly explained, see below.

Secondly, since it is known that methyl substituents on a ligand can increase the *pK_a_* value and therefore the electron donating ability thereof (see examples in Table 3 [52]), it is expected in an associative mechanism that the methyl substituted thiourea will react slightly faster than TU, as was concluded from this work on the Re^V^ system.

Thirdly, the [MO(OH_2_)(CN)_4_]^*n*−^ compounds of Tc^V^ and Re^V^ react both according to an associative mechanism or partly associative, while the Mo^IV^ and W^IV^ compounds react via a dissociative mechanism. From this, it is clear that the work done on the Mo^IV^ and W^IV^ centres, although all iso-structural *d* ^2^ species, cannot be applied directly to the Tc^V^ and Re^V^ centres, as assumed previously [13]. It also implies that the higher-oxidation state of the Tc^V^ and Re^V^centres favour the more associative activation, while the Mo^IV^ and W^IV^ could favour a dissociative activation mode.

However, even more detailed high-pressure studies, to enable construction of complete volume profiles, are required in future.

These results, along with the fact that the Tc(V) centre is much more reactive than the Re(V), is of particular relevance to nuclear medicine. In the preparation of ^99m^Tc radiopharmaceuticals or in the labelling of antibodies with ^99m^Tc, “transfer” ligands are often used to stabilize the required oxidation state, and then the actual labelling is accomplished by simple ligand substitution onto the “transfer complex” [53]. From this work, the best way of optimizing labelling conditions would be to use a S-donor transfer ligand instead of a C- or N-donor transfer ligand so that the exchange process would be dissociative in nature. This would imply that the transfer ligand, rather than the concentration of the antibody or chelating moiety attached to the antibody, would influence the reaction rate and yield a much “cleaner” reaction mixture (concentration of unlabeled antibody in solution would be low). Furthermore, the greater reactivity of Tc compared to Re must be taken into account when developing therapeutic radio-rhenium analogues to known diagnostic ^99m^Tc radiopharmaceuticals. For example, more drastic conditions are required in the preparation of ^186^Re diphosphonates (for bone imaging) than in the preparation of ^99m^Tc diphosphonates [54]. These differences in reactivities between Tc^V^ and Re^V^ centres needs to be taken into account before procedures that are available for certain technetium complexes are applied to the preparation of the rhenium analogues.

## Figures and Tables

**Table 1:** Kinetic data for the reaction between [TcO(OH_2_)(CN)_4_]^−^ and the different thiourea entering ligands; *μ* = 1.0 M (NaNO_3_), pH = 0.6. ^(a)^L.S. fits to (2); ^(b)^since small-negative intercepts were obtained in some cases, the value was fixed (= 0.00). The standard deviations reported are those from the first fits; ^(c)^L.S. fits to (1).

Parameter	*T* (°C)	NNDMTU	*T* (°C)	NMTU	*T* (°C)	TU
*k_f_* (M^−1^s^−1^)^(a)^	9.3	3.13(6)	5.7	3.26(7)	6.8	2.68(8)
	16.5	6.1(1)	15.9	5.95(6)	15.8	5.0(1)
	25.1	11.5(1)	25.1	11.38(4)	25.5	7.4(1)
*k_r_* (s^−1^)^(a), (b)^	9.3	0.00(1)	5.7	0.00(3)	6.8	0.00(2)
	16.5	0.00(4)	15.9	0.00(5)	15.8	0.00(3)
	25.1	0.00(3)	25.1	0.00(2)	25.5	0.06(2)
*pK*_*a*1_^(c)^	24.8	2.99(19)	—	—	—	—
Δ*S^#^_k__f_* (J K^−1^ mol^−1^)	—	−40(8)	—	−84(11)	—	−110(17)
Δ*H^#^_kf_* (kJ mol^−1^)	—	55(2)	—	42(3)	—	35(5)

**Table 2:** Comparative table for the kinetic data and activation parameters for the ligation reactions of [ReO(OH_2_)(CN)_4_]^−^ and [TcO(OH_2_)(CN)_4_]^−^ at 25°C. ^(a)^Refrences [37, 48], ^(b)^this work.

Parameter	Metal	NCS^−^	NNDMTU	NMTU	TU	HN_3_
*k_f_* (M^−1^ s^−1^)	Re^(a)^	0.00348(4)	0.059(2)	0.067(2)	0.0399(9)	0.0064(2)
	Tc^(b)^	22.2(3)^(a)^	11.5(4)	11.38(4)	7.4(1)	—
*k_f_* Tc/*k_f_* Re	—	6321	195	170	185	—
Δ*S^#^_k__f_* (J K^−1^ mol^−1^)	Re^(a)^	−11(6)	−119(40)	−125(10)	−95(3)	−87(6)
	Tc^(b)^	−9(12)	−40(8)	−84(11)	−110(17)	—
Δ*H^#^_k__f_* (kJ mol^−1^)	Re^(a)^	17.4(1.9)	45(11)	42(3)	52(1)	60(2)
	Tc^(b)^	62(4)	55(2)	42(3)	35(5)	—
Δ*V^#^_k__f_* (cm^3^ mol^−1^)	Re^(b)^	−1.7(3)	—	—	−22.1(9)	—
	Tc^(b)^	−3.5(3)	−14(1)	—	−6.0(5)	—

**Table 3:** Comparison between *pK*_*a*_ values of methyl substituted and unsubstituted compounds [52].

Compound	*pK*_*a*1_	Compound	*pK*_*a*1_
Cinnamic acid	4.44	*P*-Methylcinnamic acid	4.56
Malonic acid	2.83	Methylmalonic acid	3.07
Acetic acid	4.75	Trimethylacetic acid	5.03
Pyridine	5.25	2,4-Dimethylpyridine	6.57
Methylamine	10.66	Dimethylamine	10.73
